# USE OF SCORE AND CEREBROSPINAL FLUID LACTATE DOSAGE IN DIFFERENTIAL DIAGNOSIS OF BACTERIAL AND ASEPTIC MENINGITIS

**DOI:** 10.1590/1984-0462/;2017;35;4;00010

**Published:** 2017

**Authors:** Frederico Ribeiro Pires, Andréia Christine Bonotto Farias Franco, Alfredo Elias Gilio, Eduardo Juan Troster

**Affiliations:** a Hospital Israelita Albert Einstein, São Paulo, SP, Brasil.; b Departamento de Pediatria da Faculdade de Medicina da Universidade de São Paulo, São Paulo, SP, Brasil.

**Keywords:** Bacterial meningitis score, Meningitis, Lactate, Escore de meningite bacteriana, Meningite, Lactato

## Abstract

**Objective:**

To evaluate Bacterial Meningitis Score (BMS) on its own and in association with Cerebrospinal Fluid (CSF) lactate dosage in order to distinguish bacterial from aseptic meningitis.

**Methods:**

Children diagnosed with meningitis at a tertiary hospital between January/2011 and December/2014 were selected. All data were obtained upon admission. BMS was applied and included: CSF Gram staining (2 points); CSF neutrophil count ≥1,000 cells/mm^3^ (1 point); CSF protein ≥80 mg/dL (1 point); peripheral blood neutrophil count ≥10,000 cells/mm^3^ (1 point) and seizures upon/before arrival (1 point). Cutoff value for CSF lactate was ≥30 mg/dL. Sensitivity, specificity and negative predictive value of several BMS cutoffs and BMS associated with high CSF lactate were evaluated for prediction of bacterial meningitis.

**Results:**

Among 439 eligible patients, 94 did not have all data available to complete the score, and 345 patients were included: 7 in bacterial meningitis group and 338 in aseptic meningitis group. As predictive factors of bacterial meningitis, BMS ≥1 had 100% sensitivity (95%CI 47.3-100), 64.2% specificity (58.8-100) and 100% negative predictive value (97.5-100); BMS ≥2 or BMS ≥1 associated with high CSF lactate also showed 100% sensitivity (47.3-100); but 98.5% specificity (96.6-99.5) and 100% negative predictive value (98.3-100).

**Conclusions:**

2 point BMS in association with CSF lactate dosage had the same sensitivity and negative predictive value, with increased specificity for diagnosis of bacterial meningitis when compared with 1-point BMS.

## RESUMO

**Objetivo:**

Avaliar o Escore para Meningite Bacteriana (EMB) isolado e associado ao valor do lactato no líquor para diferenciar meningite bacteriana (MB) e meningite asséptica (MA).

**Métodos:**

Foram selecionadas crianças com meningite atendidas em hospital terciário privado entre janeiro de 2011 e dezembro de 2014. Os dados foram obtidos na admissão. Utilizou-se o EMB com: coloração de Gram no líquor (2 pontos); neutrófilos no líquor ≥1.000 células/mm^3^ (1 ponto); proteína no líquor ≥80 mg/dL (1 ponto); neutrófilos no sangue periférico ≥10.000 células/mm^3^ (1 ponto); e convulsão durante/antes da chegada (1 ponto). Analisou-se também o lactato no líquor (elevado: ≥30 mg/dL). Avaliaram-se sensibilidade, especificidade e valor preditivo negativo de diversos valores de corte do EMB e do EMB associado ao lactato elevado para prever MB.

**Resultados:**

Dos 439 pacientes elegíveis, 94 não tinham todos os dados necessários para o escore, sendo 345 pacientes selecionados: 7 no grupo de MB e 338 no de MA. Como preditivos de MB, o EMB ≥1 mostrou sensibilidade de 100% (intervalo de confiança de 95% - IC95% 47,3-100), especificidade de 64,2% (58,8-100) e valor preditivo negativo de 100% (97,5-100), enquanto o EMB ≥2 ou EMB ≥1 associado a lactato liquórico ≥30 mg/dL mostrou sensibilidade de 100% (47,3-100), especificidade de 98,5% (96,6-99,5) e valor preditivo negativo de 100% (98,3-100).

**Conclusões:**

O EMB com 2 pontos associado à dosagem de lactato no líquor manteve a sensibilidade e o valor preditivo negativo, ao passo que aumentou a especificidade para identificar meningites bacterianas em relação à utilização do EMB com 1 ponto.

## INTRODUCTION 

Upon initial care of children with meningitis, distinction between bacterial and aseptic forms is fundamental.^1^ For bacterial meningitis (BM), the immediate course of antibiotic therapy and hospitalization are paramount, whereas in aseptic meningitis (AM, usually viral) only supportive measures are necessary.^1^ ^,^ ^2^ ^,^ ^3^ ^,^ ^4^ ^,^ ^5^ ^,^ ^6^ ^,^ ^7^However, this distinction is not always easy in daily practice, especially in cases of viral meningitis with predominance of neutrophils in first cerebrospinal fluid (CSF) collection. For this reason, many children with viral meningitis are hospitalized and receive antibiotics treatment until cultures are assessed, which may take a few days.^8^ ^,^ ^9^ ^,^ ^10^As there is a need to reduce hospitalizations and the unnecessary use of antibiotics, two scores were proposed: Bacterial Meningitis Score (BMS) was proposed by Nigrovic et al.^11^ and has shown sensitivity and negative predictive value close to 100%; Meningitest, proposed by European authors,^12^ was shown to be less specific when compared BMS. The score of Nigrovic et al.^11^, shown in Table 1, uses one clinical and four laboratorial criteria. Each criterion is assigned one point, while Gram criterion is assigned two. If all of them test negative, sensitivity and negative predictive value to rule out BM are 100%.^11^

**Table 1:** Bacterial Meningitis Score*.

Bacterial Meningitis Score predictors	Criteria
CSF Gram stain	Positive (2 points)
CSF absolute neutrophils	≥1,000 cells/mm^3^ (1 point)
CSF protein	≥80 mg/dL (1 point)
Peripheral blood absolute neutrophils	≥10,000 cells/mm^3^ (1 point)
Seizure	Upon or right after arrival (1 point)

CSF: cerebrospinal fluid; *from Nigrovic et al.^11^.

Several studies have emerged after the publication of the study by Nigrovic et al.^11^ to evaluate this score.^13^ ^,^ ^14^ In 2012, the same group of authors published a meta-analysis that confirmed the high sensitivity and high negative predictive value of the score.^2^ In Brazil, Mekitarian Filho and coworkers found excellent results after applying the score in children with meningitis assisted at the University Hospital of Universidade de São Paulo (HU/USP), with 100% sensitivity and negative predictive value.^15^ In addition to scores, one can also use CSF lactate dosage in order to distinguish MB from MA. Cutoff points in studies that showed the best sensitivity and specificity values are not well established, ranging from 2.1 to 4.4 mmol/L; however, a recent study conducted in Brazil used 30 mg/dL.^16^

The only Brazilian study that employed BMS was performed at one institution only. Therefore, studies comprising more healthcare centers are necessary so that results can be validated. Thus, our primary goal was to evaluate the discriminatory power of BMS to differentiate MB and MA, in the context of a Brazilian tertiary, private hospital. Secondary purpose was to verify CSF lactate also for the distinction between bacterial and aseptic meningitis.

## METHOD

All children aged 1 month and 14 years who were assisted at the emergency service of Hospital Israelita Albert Einstein (HIAE, São Paulo, SP) between January 1, 2011 and December 31, 2014, with meningitis-related International Classification of Diseases (ICD) (A87, A87.0-9, G00, and G00.0-9) were initially selected for the study.

This is a retrospective cohort study, carried out by medical records data analysis and search for laboratory tests in the institution’s website. All data were collected at admission. Two authors (FRP and ACBFF) were responsible for collecting and compiling data.

Inclusion criteria were: age between 1 month and 14 incomplete years on the day of medical assistance; Diagnosis of meningitis with CSF leukocyte >9 cells/µL (considering leukocytes-erythrocytes ratio of 1:500 for puncture accident). Exclusion criteria were: hemodynamic instability, evidence of cerebral herniation, presence of ventricular shunt or recent neurosurgery; previous epilepsy occurrence, immunosuppression; other bacterial infection requiring parenteral antibiotics or having received antibiotics 72 hours prior to lumbar puncture.

The variables analyzed were gender, age, occurrence of seizures, absolute neutrophil and C-reactive protein (CRP), assessment of CSF glucose, lactate, neutrophils, protein and Gram, as well as blood and CSF cultures. All exams were collected at admission. Differential cytology for CSF was performed with microscopic counting after cytospin concentration and panchromatic staining. Biochemical features, lactate included, were processed by a dry-chemistry automated method (Fusion^®^).

The outcomes analyzed were presence of BM or AM. In the bacterial meningitis group, children who had positive CSF culture or pleocytosis (>9/mm^3^) associated with positive blood culture for bacterial pathogen were considered. The aseptic meningitis group comprised all other children, who did not fit the standards described.

The study sample (345 patients) was obtained for convenience in a 4-year period, which is sufficient for the estimation of a 95% confidence interval (95%CI) and 65% accuracy measure, with 5% sampling error. As both primary and secondary objectives of the study did not involve testing hypotheses, sample statistical power estimation was not applied.

In statistical analysis, the patients’ gender was described in groups as absolute frequencies and percentages and compared with by Fisher’s exact test. Numerical variables were described as medians and quartiles and compared with by the Mann-Whitney test. BMS power to distinguish children with BM and AM was assessed by measuring accuracy, sensitivity, specificity, positive and negative predictive values; kappa coefficient was also used to compare agreement between score and cultures in BM scenario, all measures being accompanied by respective 95% intervals. The calculations were made in compliance with recommendations by Altman^17^ and aided by R packages 3.1.3 (R Foundation for Statistical Computing, Vienna, Austria) and Microsoft Excel 2010 (Microsoft Redmond, USA). The significance level adopted in comparisons was 5%.

The project was approved by the Ethics Committee of HIAE and Platforma Brasil, under CAAE consubstantiate opinion: 46335015.2.0000.0071.

## RESULTS 

A total of 503 patients admitted to HIAE (SP) between 2011 and 2014 were identified and met the aforementioned inclusion criteria. Among these, 64 were excluded due to the following conditions: previous use of antibiotic (n=26); no diagnosis of meningitis (n=29); two previous hospital admissions (n=8); previous occurrence of epilepsy (n=1). Among 439 children eligible for examination, 94 did not perform all tests required for BMS analysis, 90 were not submitted to blood count, and four did not have CSF Gram staining, so they were excluded afterwards. Thus, 345 patients fulfilled all prerequisites and were selected for the study (Flowchart 1): seven were allocated in BM group, and 338 in AM group.

**Flowchart 1:**
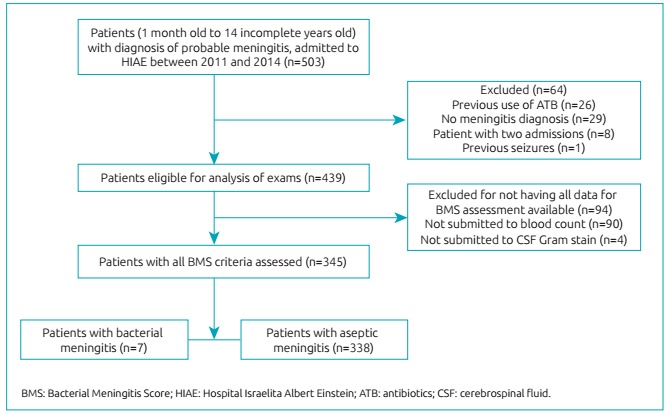
Patients enrollment.

The microorganisms isolated in cultures of patients in BM group were: *Streptococcus pneumoniae* (five cases), *Neisseria meningitidis* (one case), and *Enterococcus faecalis* (one case). In AM group, 292 had CSF investigated for polymerase chain reaction enterovirus and 237 tested positive (81%).

When comparing groups, no evidence of difference in age, gender, or blood neutrophils count was found (Table 2). The BM group had a higher CSF lactate value (60.7 *versus* 15.5 mg/dL in AM), CSF neutrophils count (1,866 *versus* 32.5/mm^3^ in AM), blood PCR (23.3 *versus* 1.53 mg/L in AM) and CSF protein (83 *versus* 30 mg% in AM), as shown in Table 2.

**Table 2:** Comparison between groups with bacterial and aseptic meningitis.

	Aseptic meningitis (n=338)	Bacterial meningitis (n=7)	p-value
Gender*			
Female	138 (40.8)	2 (28.6)	0.705
Male	200 (59.2)	5 (71.4)	
Age (years)^&^	5.1 [3.6-7.1]	3.2 [0.7-6.3]	0.266
CSF lactate (mg/dL)^&^	15.5 [13.5-17.4]	60.7 [46.6-71.3]	<0.001
CSF neutrophils (n/mm^3^)^&^	32.5 [10.0-94.3]	1866.0 [939.0-2573.0]	<0.001
Blood neutrophils (n/mm^3^)^&^	8524.0 [6065.0-11086.8]	18144.0 [8293.5-24173.0]	0.066
Blood CRP (mg/L)^&^	1.5 [0.7-2.8]	23.3 [17.0-30.7]	<0.001
CSF protein (mg%)^&^	30.0 [23.0-40.8]	83.0 [73.0-275.0]	<0.001

CSF: cerebrospinal fluid; CRP: C-reactive protein; *Expressed as n (%); ^**&**^ Expressed as median (interquartile range).

In BMS analysis, 1 point was the cutoff for differential diagnosis of MB and MA; that is, if the patient scored 1 or more in BMS, it should be treated as probable MB. Results were: 100% sensitivity (95%CI 47.3-100), 64.2% specificity (95%CI 58.8-69.3), 100% negative predictive value (95%CI 97.5-100), and kappa coefficient for score and CSF culture of 0.07 (95%CI -0.07 -0.2).

When using the 2 as cutoff point in BMS and considering patients who scored 2 or more as BM, the same 100% sensitivity (95%CI 47.3-100) and negative predictive value were obtained (95%CI 98.3-100), but specificity was increased to 98.8% (95%CI 97.0-99.7), and kappa coefficient for CSF score and culture was 0.77 (95%CI 0.55-0.99) (Table 3).

**Table 3:** Scores comparison.

Measures	BMS≥1 % (95%CI)	BMS≥2 % (95%CI)	Lactate≥30 mg/dL % (95%CI)	BMS+lactate* % (95%CI)
Sensitivity	100.0 (47.3-100.0)	100.0 (47.3-100.0)	85.7 (42.1-99.6)	100.0 (47.3-100.0)
Specificity	64.2 (58.8-69.3)	98.8 (97.0-99.7)	99.1 (97.4-99.8)	98.5 (96.6-99.5)
Accuracy	64.9 (59.6-70.0)	98.8 (97.1-99.7)	98.8 (97.1-99.7)	98.6 (96.7-99.5)
Positive predictive value	5.5 (2.2-10.9)	63.6 (30.8-89.1)	66.7 (29.9-92.5)	58.3 (27.7-84.8)
Negative predictive value	100.0 (97.5-100.0)	100.0 (98.3-100.0)	99.7 (98.4-100.0)	100.0 (98.3-100.0)
Kappa coefficient	0.07 (-0.07-0.20)	0.77 (0.55-0.99)	0.74 (0.49-0.99)	0,73 (0,49-0,96)

BMS: bacterial meningitis score; *Considered bacterial meningitis if BMS≥2 or BMS≥1 associated with lactate in CSF ≥30 mg/dL.

When using CSF lactate dosage with 30 mg/dL as cutoff value for BM or AM diagnosis, results were: 85.7% sensitivity (95%CI 42.1-99.6), 99.1% specificity (95%CI 97.4-99.8), 99.7% negative predictive value (95%CI 98.4-100.0), and kappa coefficient for CSF score and culture of 0.74 (95%CI 0.49-0.99).

Combining 2-point and 1-point BMS results and CSF lactate ≥30 mg/dL, findings were: 100% sensitivity (95%CI 47.3-100.0), 98.5% specificity (95%CI 96.6-99.5), 100% negative predictive value (95%CI 98.3-100.0), and kappa coefficient for CSF score and culture of 0.73 (95%CI 0.49 -0.96).

## DISCUSSION

When analyzing 1-point BMS predictive value for BM, results were 100% sensitivity and negative predictive value, but specificity was low (64.2%), which is in accordance with what has already been reported by other authors.^11^ ^,^ ^12^ ^,^ ^13^ ^,^ ^14^ ^,^ ^15^In the present study, the wide range of sensitivity confidence (47 to 100%) draws attention. Such amplitude results from the small number of BM cases in our sample, compared to the large number of AM cases. Moreover, kappa coefficient was low in this analysis, ^17^ that is, the agreement between score and the gold standard method (culture) is reduced, indicating that the BMS may not be the best choice for the population studied.

In the analysis of 2-point BMS, we found 100% sensitivity and a negative predictive value, besides specificity being raised to 98.8%, which would generate low hospital admission rate, but still counting BM hospitalization cases. However, because of the small number of BM cases compared to AM, care should be taken when interpreting such data, since Nigrovic et al.^11^ reported an 87% sensitivity when the cutoff point was increased to 2. That is, 13% of children with BM were probably at risk of having been discharged from initial care if BMS was 2 points in the American study.

For CSF lactate with ≥30 mg/dL cutoff, we found 85.7% sensitivity, 99.1% specificity, and 99.7% negative predictive value. Our results are in agreement with the literature, that is, this is a very specific but not very sensitive criterion.^16^ Therefore, using this method alone poses the risk of non-admitting BM patients.

Since 2-point BMS did not individually demonstrate 100% sensitivity in another study^11^ and because CSF lactate alone was also a specific but not very sensitive criterion, we proposed the union of both parameters. When considering 2 or more points or 1-point BMS associated with CSF lactate value greater or equal to 30 mg/dL as predictive of BM, sensitivity was100%, specificity was 98.5%, and negative predictive value was 100%. Those who did not meet these criteria were considered AM. Kappa coefficient of 0.73 shows good concordance^17^ between this new score and confirmation of BM. Instead of applying only 1-point BMS, as already proposed in other studies, the advantage of this new approach is the significant increase in specificity, without giving up high sensitivity.

In our study, the frequency of BM was 2% (95%CI 0.8-4.1). This data differs a lot from those obtained by Mekitarian Filho et al., who reported 9% of BM cases when analyzing the clinical picture of 547 children between 2001 and 2012 in a public hospital of São Paulo.^15^ Differences in socioeconomic status and, especially, in vaccination history, may explain such results.^18^

Our study had, though, a few limitations. Being a retrospective survey may cause important information not to be obtained, especially regarding subjects’ general state and toxemia, or their vaccination history. Also, as the study was performed in one institution alone, it reflects the reality of that place solely. In addition, the number of BM cases found was small, and this may have impaired the reproducibility of the modified score. The number of BM cases also explains the broad values in confidence intervals for sensitivity in all scores evaluated.

Bearing such limitations in mind, this is the first Brazilian study to analyze accuracy of BMS in a private tertiary service which showed promising results like those reported in the literature and also confirmed previous findings of a Brazilian study conducted in a public hospital.^15^ The use of a new criterion for initial differentiation between BM and AM by combining CSF lactate and BMS is promising and had great sensitivity and specificity in our series. However, further studies are needed, especially in institutions with large numbers of BM cases, so that the accuracy of this standard can be assessed and new tools can be provisioned for the care of children with meningitis.
